# 660. *In vitro* activity of cefiderocol against difficult-to-treat resistance European Gram-negative bacterial pathogens from the multi-national sentinel surveillance study, SENTRY in 2020 and 2021

**DOI:** 10.1093/ofid/ofac492.712

**Published:** 2022-12-15

**Authors:** Anne Laurence Santerre Henriksen, Christopher M Longshaw, Dee Shortridge, Jennifer M Streit, Miki Takemura, Yoshinori Yamano

**Affiliations:** Maxel Consulting ApS, Jyllinge, Sjelland, Denmark; Shionogi B.V., London, England, United Kingdom; JMI Laboratories, North Liberty, Iowa; JMI Laboratories, North Liberty, Iowa; Shionogi & Co., Ltd, Toyonaka, Osaka, Japan; Shionogi & Co., Ltd., Toyonaka, Osaka, Japan

## Abstract

**Background:**

Difficult-to-treat resistance (DTR) organisms are defined as non-susceptible to all first-line high-efficacy, low-toxicity antibiotics (penicillins, cephalosporins, carbapenems and fluoroquinolones), leaving physicians with limited treatment options. Cefiderocol is a parenteral siderophore cephalosporin with potent activity against aerobic Gram-negative pathogens, including carbapenem-resistant strains. We evaluated the *in vitro* activity of cefiderocol and comparators against DTR pathogens collected in Europe by the SENTRY surveillance study in 2020 and 2021.

**Methods:**

A total of 11434 clinical isolates of Gram-negative bacilli were systematically collected from 16 EU countries, Israel and Turkey in 2020 and 2021. Minimum inhibitory concentrations (MICs) were determined by broth microdilution for a panel of twenty-two antibiotics according to CLSI guidelines. All antibiotics were tested in cation-adjusted Mueller-Hinton broth (CAMHB) except for cefiderocol, for which iron-depleted CAMHB was used. Susceptibility was determined according to CLSI breakpoints, and DTR pathogens were defined as being resistant to cefepime, ceftazidime ceftriaxone, imipenem, meropenem, ciprofloxacin and levofloxacin according to CLSI breakpoints.

**Results:**

Among 11434 Gram-negative isolates collected in 2020 and 2021, 792 (7.0%) were resistant to all 1^st^ line therapy including cephalosporins, carbapenems and fluoroquinolone and could be defined as DTR. DTR was most frequently observed in *Acinetobacter* spp. (530/931, 56.9%), Enterobacterales (201/7739, 2.6%) and *Pseudomonas aeruginosa* (61/2440, 2.5. Based on CLSI breakpoints, cefiderocol was the most active antibiotic tested against DTR-*Acinetobacter* spp. (MIC_90_= 2mg/L, 97.4% susceptibility). Ampicillin/sulbactam was active in less than 1% of the DTR-*Acinetobacter* spp isolates. None of the drugs recommended by the IDSA for the treatment of resistant Gram-negative infections were as potent as cefiderocol (**Table 1**).

**Figure:**
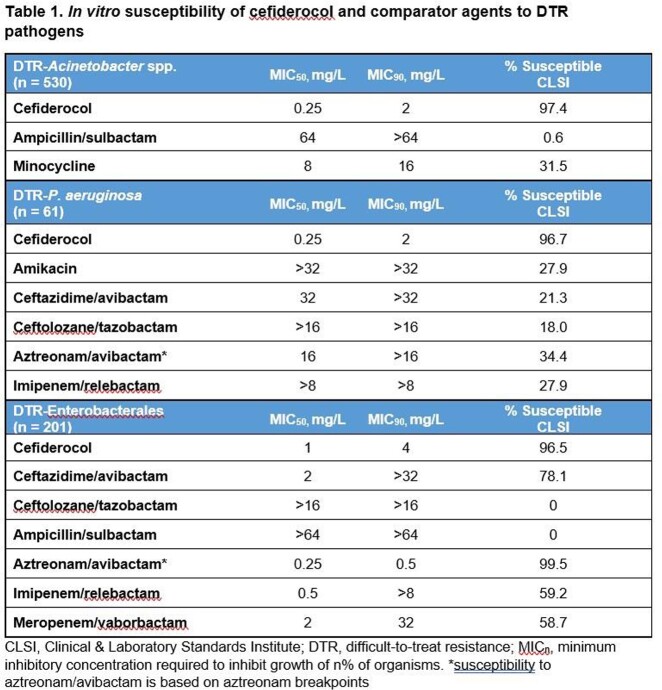

**Conclusion:**

Cefiderocol was the only treatment option with demonstrated in vitro activity against more than 95% of all the tested DTR Gram-negative pathogens with limited treatment options.

**Disclosures:**

**Anne Laurence Santerre Henriksen, PhD**, Shionogi: Contractor|UTILITY therapeutics Ltd: Advisor/Consultant **Christopher M. Longshaw, PhD**, Shionogi: Employee **Dee Shortridge, PhD**, AbbVie: Grant/Research Support|JMI Laboratory: Employee|Melinta: Grant/Research Support|Menarini: Grant/Research Support|Shionogi: Grant/Research Support **Jennifer M. Streit, BS, MT(ASCP)**, Cidara: Grant/Research Support|GSK: Grant/Research Support|Melinta: Grant/Research Support|Shionogi: Grant/Research Support **Miki Takemura, n/a**, Shionogi: Employee **Yoshinori Yamano, PhD**, Shionogi: Employee.

